# PCA-based population structure inference with generic clustering algorithms

**DOI:** 10.1186/1471-2105-10-S1-S73

**Published:** 2009-01-30

**Authors:** Chih Lee, Ali Abdool, Chun-Hsi Huang

**Affiliations:** 1 Computer Science and Engineering Department, University of Connecticut, Storrs, CT 06269, USA

## Abstract

**Background:**

Handling genotype data typed at hundreds of thousands of loci is very time-consuming and it is no exception for population structure inference. Therefore, we propose to apply PCA to the genotype data of a population, select the significant principal components using the Tracy-Widom distribution, and assign the individuals to one or more subpopulations using generic clustering algorithms.

**Results:**

We investigated K-means, soft K-means and spectral clustering and made comparison to STRUCTURE, a model-based algorithm specifically designed for population structure inference. Moreover, we investigated methods for predicting the number of subpopulations in a population. The results on four simulated datasets and two real datasets indicate that our approach performs comparably well to STRUCTURE. For the simulated datasets, STRUCTURE and soft K-means with BIC produced identical predictions on the number of subpopulations. We also showed that, for real dataset, BIC is a better index than likelihood in predicting the number of subpopulations.

**Conclusion:**

Our approach has the advantage of being fast and scalable, while STRUCTURE is very time-consuming because of the nature of MCMC in parameter estimation. Therefore, we suggest choosing the proper algorithm based on the application of population structure inference.

## Background

Population structure inference is the problem of assigning each individual in a population to a cluster, given the number of clusters. When admixture is allowed, each individual can be assigned to more than one cluster along with a membership coefficient for each cluster. Population structure inference has many applications in genetic studies. Some obvious applications include grouping individuals, identifying immigrants or admixed individuals, and inferring demographic history. Moreover, it also serves as a preprocessing step in stratified association studies to avoid spurious associations [1].

The association between a marker and a locus involved in disease causation has been the object of numerous studies. In a case-control study, it is possible that the samples or patients are drawn from two or more different populations but the population structure is not observed or recorded. Suppose that an allele of a marker appears significantly more frequently in the case than in the control group, we might come to the conclusion that this allele is associated with the disease. However, we have to rule out the possibility that most of the samples in the case group are from a specific population and this allele happens to be the prevalent one at the marker. Therefore, inferring population structure before association studies allow us to avoid this problem, lowering the false positive rate.

Software STRUCTURE is widely used in population structure inference. It is specifically designed for genotype data and approaches the problem by careful modelling of allele frequencies, origins of alleles of individuals and origins of individual genomes. As described in Section **Methods**, for a genotype dataset of *m *diploid individuals and *n *biallelic markers, STRUCTURE estimates 2*Kn *+ *Km *+ 2*mn *parameters using Markov Chain Monte Carlo (MCMC), where *K *is the number of clusters. Inferring population structure using STRUCTURE is, therefore, very time-consuming since it has to handle large datasets consisting of thousands of individuals genotyped at hundreds of thousands of loci. Therefore, we propose an alternative approach to dealing with this problem.

From the perspective of machine learning, when dealing with high-dimensional data, it is natural to preprocess the data with dimension reduction and feature selection techniques. Principal component analysis (PCA) is a technique of dimension reduction. The importance of a principal component (PC) is proportional to the corresponding eigenvalue, which is the variance of data projected onto this component. Deciding the number of PCs to be kept for subsequent analyses is not a trivial problem. Fortunately, Johnstone [2] showed that with suitable normalization, for large *m *and *n*, the distribution of the largest eigenvalue *λ*_1 _is approximately a Tracy-Widom (TW) distribution [3]. Patterson *et al*. [4] applied PCA to real and simulated population genotype data with more than one underlying subpopulation. It is shown that, when the genotype data is projected onto a significant PC, the means of the subpopulations are also significantly different according to an ANOVA test. These empirical results indicate the potential of PCA and the TW distribution in discovery of population structure. Therefore, we propose to perform dimension reduction on genotype data using PCA and apply generic clustering algorithms to infer population structure.

In this paper, we base our study on PCA and investigate three generic clustering algorithms – K-means, soft K-means and spectral clustering algorithms. The results are then compared with those generated by STRUCTURE. We introduce the data, clustering algorithms and evaluation metric in Section **Methods**. Comparisons and analyses of results are given in Section **Results and discussion**. Finally, we give the concluding remarks in Section **Conclusions**.

## Methods

### Data

In this study, we use both real and simulated data to evaluate the performance of clustering algorithms. The real data is obtained from the Human Genome Diversity Project-Centre d'Etude du Polymorphisme Humain (HGDP-CEPH) Human Genome Diversity Panel [5], which contains genotypes of 1,064 individuals sampled from 51 populations. The version 2.0 of the HGDP-CEPH database contains genotypes for 4,991 markers and 4,154 biallelic ones are used in our study. Two subsets of individuals are constructed from the 1,064 ones. One subset encompasses all the 258 individuals in Europe and Middle East, which are geographically close, and we refer to it as the close dataset. The other subset consists of all the 739 individuals in Africa, Central South Asia, East Asia and Europe, which are geographically far apart from each other, and we refer to it as the distant dataset.

**Figure 1:**
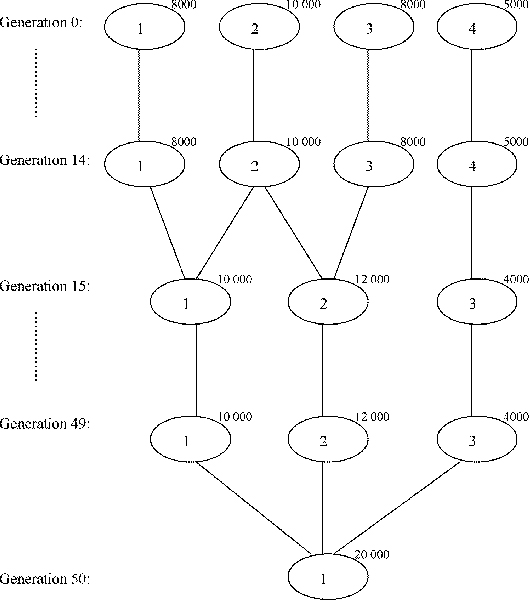
**The demography used in simulating the fourth dataset**. Generation 0 represents the current generation, while generation *g *represents *g *generations back in time.

The simulated data is generated using software GENOME, a coalescent-based simulator written by Liang *et al*. [6]. The parameters are set to mimic the real data from HGDP-CEPH. The number of chromosomes or independent regions is set to 22 since there are 22 autosomal chromosomes in human. Each chromosome has 100 10,000-base fragments, simulating linkage disequilibrium within fragments. The recombination rate between two consecutive fragments is set to 0.01 to simulate the length of human genome. The number of markers per chromosome is set to a fixed number of 250, so the number of markers for each individual is 5,500. We use four simulated datasets in this study. Three of them contain individuals sampled from independent populations. The fourth dataset is generated according to a simple demography shown in Figure 1. The details are summarized in Table 1.

**Table 1:** Details of the first three simulated datasets

Set	#idvs	#pops	#idvs from each pop
1	300	3	100 100 100
2	400	4	100 100 100 100
3	500	4	50 100 150 200
4	620	4	160 200 160 100

### Principal component analysis

Principal component analysis (PCA) is a technique of dimension reduction. Given *m *samples and *n *markers or variables, the *m *samples can be represented as a *m *× *n *matrix **X**. We further assume that the sample mean of each marker is 0, i.e., $\sum_{i=1}^{m}X_{ij}=0$. Using another basis of *n *vectors or axes, represented as column vectors of **P**, we can project the samples onto the new axes and obtain another *m *× *n *matrix **Y **= **XP**. PCA finds a **P **such that the sample covariance matrix of the *n *new variables is a diagonal matrix. That is,

$$\sum_{Y}=\frac{1}{m}Y^{T}Y=\frac{1}{m}{\left(XP\right)}^{T}XP=\frac{1}{m}P^{T}X^{T}XP=P^{T}\sum_{X}P=D,$$

where **D **is a diagonal matrix, **Σ**_**X **_and **Σ**_**Y **_are the sample covariance matrices of the original and new *n *variables, respectively. **P **can be obtained by the eigen decomposition of **Σ**_**X**_. Therefore, PCA is very simple and easy to implement.

In this study, we use the software SMARTPCA by Patterson *et al*. [4]. SMARTPCA is specifically designed for genotype data and it offers options addressing issues such as linkage disequilibrium (LD) in analyzing genotype data. Patterson *et al*. [4] showed that the presence of LD in data distorts the distribution of eigenvalues, which makes selecting PCs according to the TW statistics meaningless. Therefore, we follow the suggestion and turn on the option to replace the values of each marker with the residuals from a multivariate regression without intercept on the 2 preceding markers. After PCA, we keep those PCs with *p*-values smaller than 5% for subsequent cluster analyses. Since STRUCTURE accepts only genotype data, the input to STRUCTURE is not processed with PCA.

### Clustering algorithms

In this study, we investigate three generic clustering algorithms – K-means, soft K-means and spectral clustering algorithms. In order to compare these generic clustering algorithms to algorithms designed specifically for population structure inference, we also run STRUCTURE on the datasets. We briefly introduce the three generic clustering algorithms and STRUCTURE in the folowing subsections.

#### K-means

The K-means algorithm is an iterative descent algorithm that minimizes the within-cluster sum of squares (WSS) given the number of clusters *K*.

(1) $$W_{K}=\sum_{i=1}^{K}\sum_{j\in C_{i}}{\left\|x_{j}-\mu_{i}\right\|}^{2},$$

where **x**_*j *_is the feature vector representing sample *j*, *μ*_*i *_is the center of cluster *i*, and *C*_*i *_is the set of samples in cluster *i*. We use the implementation of a variant by Hartigan and Wong [7] embedded in the R Language.

#### Soft K-means

The soft K-means algorithm assumes that samples follow a mixture of *K *multivariate Gaussian distributions $\sum_{k=1}^{K}\delta_{k}\text{N}(\mu_{k},\sum_{k}),$, where $\sum_{k}\delta_{k}=1$; *μ*_*k *_and **Σ**_*k *_are the mean and covariance matrix for the *k*^th ^Gaussian distribution. Therefore, given the number of clusters *K*, the algorithm estimates the parameters *θ *= (*δ*_1_,...,*δ*_*K*_, *μ*_1_, **Σ**_1_,...,*μ*_*K*_, **Σ**_*K*_) using the Expectation-Maximization Algorithm, while the unobserved latent variables are the labels of samples. In this study, we use MCLUST Version 3 [8] for R Language, which offers a wide selection of covariance matrix models.

#### Spectral clustering

The spectral clustering algorithm is based on the weighted graph partitioning problem. Considering a graph of *m *nodes, each node represents a sample and the weight on the edge between two nodes is the similarity between the two samples. We define the total similarity between two clusters *A*, *B *as

$$\text{Sim}(A,B)=\sum_{i\in A}\sum_{j\in B}S_{ij},$$

where **S **is a *m *× *m *similarity matrix. Given the number of clusters *K*, we want to find a partition C* such that the following objective function is minimized.

(2) $$C*=\arg\underset{C}{\min}\sum_{k=1}^{K}\frac{\text{Sim}(C_{k},\cup_{i=1,i\neq k}^{K}C_{i})}{\text{Sim}(C_{k},\cup_{i=1}^{K}C_{i})}$$

**Figure 2:**
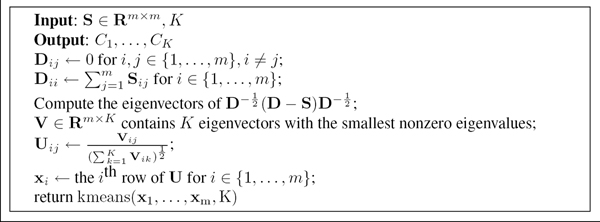
The spectral clustering algorithm.

Equation 2 can be expressed as follows.

(3) $$E*=\arg\underset{E}{\min}\sum_{k=1}^{K}\frac{e_{k}^{T}(D-W)e_{k}}{e_{k}^{T}De_{k}},$$

where **E **= (**e**_1_,...,**e**_*K*_) is a *m *× *K *indicator matrix and **D **is a *m *× *m *diagonal degree matrix. The *i*^th ^element of **e**_*k *_is 1 if sample *i *is in cluster *k*. Otherwise, it is 0. $D_{ii}=\sum_{j=1}^{m}S_{ij}$. Since finding the optimal **E **is NP-hard, spectral clustering solves the minimization problem by allowing the entries of **E **to have real values. This amounts to finding the *K *eigenvectors of $D^{-\frac{1}{2}}(D-S)D^{-\frac{1}{2}}$ with the smallest nonzero eigenvalues. We implemented the algorithm, described in Figure 2, proposed by Ng *et al*. [9] in R. In the last line of the algorithm, one can use any algorithm to perform the clustering. Therefore, we investigate K-means and soft K-means, producing two variants of the spectral clustering algorithm. In this study, we use a radial basis function to calculate the similarity between two samples.

(4) **S**_*ij *_= exp(-*γ*||**x**_*i *_- **x**_*j*_||^2^),

where *γ *is a constant.

#### STRUCTURE

Given the number of clusters *K *and genotype data **X**, STRUCTURE [10] models the population structure with three vectors of parameters – **Q**, **Z **and **P**. The genotype data and parameter vectors contain the following elements.

$$\begin{array}{ccc} x_{l}^{\left(i,a\right)} & = & \text{allele copy }a\text{ of individual }i\text{ at locus }l;\\ q_{k}^{\left(i\right)} & = & \text{proportion of individual }i\text{'s genome}\\ & & \text{that originated from population }k\text{;}\\ z_{l}^{\left(i,a\right)} & = & \text{population origin of allele copy }x_{l}^{\left(i,a\right)}\text{;}\\ p_{klj} & = & \text{frequency of allele }j\text{ at locus }l\text{ in}\\ & & \text{population }k. \end{array}$$

In diploid organisms, there are two copies of alleles at each locus on an autosomal chromosome, and hence *a *∈ {1, 2}. The probability model for (**X**, **Z**, **P**, **Q**) is described by the following equations:

$$\begin{array}{c} \text{P}(x_{l}^{\left(i,a\right)}=j|Z,P,Q)=p_{z_{l}^{{\left(i,a\right)}_{lj}}};\\ P(z_{l}^{\left(i,a\right)})=k|P,Q)=q_{k}^{\left(i\right)};\\ p_{kl}\sim \text{D}(\lambda_{1},\ldots ,\lambda_{J_{l}}), \end{array}$$

where D(·) is the Dirichlet distribution, *J*_*l *_is the number of alleles at locus *l*, and *λ*_1 _= ... = $\lambda_{J_{l}}$ = 1.0, giving a uniform distribution on the allele frequencies;

**q**^(*i*) ^~ D(*α*,...,*α*),

where D(·) is again the Dirichlet distribution and *α *∈ [0, 10] is uniformly distributed. The estimates of **Z**, **P**, and **Q **are obtained by sampling **Z**, **P**, **Q **from the posterior distribution P(**Z**, **P**, **Q**|**X**) using a MCMC algorithm. In this study, the burn-in length is set to 5,000 and another 5,000 samples are collected after burn-in for parameter estimation.

### Inferring the number of clusters

The number of clusters is always an important issue in cluster analysis. As a model-based algorithm, STRUCTURE estimates the number of clusters *K *using the posterior distribution of *K*

P(*K*|**X**) ∝ P(**X**|*K*)P(*K*),

where **X **denotes the genotype data. In this study, we investigate two methods for selecting the number of clusters. One is a distance-based generic method using the gap statistic proposed by Tibshirani *et al*. [11]. The other is by using the Bayesian Information Criterion (BIC) [12] as the model selection criterion with the soft K-means clustering algorithm. We briefly introduce the two methods in the following paragraphs. The gap statistic is a heuristic method based on the WSS given in Equation 1. Given the number of clusters, we expect smaller WSS in a dataset that has clusters than in one that do not. Therefore, the gap statistic is defined as follows.

(5) $$\text{Gap}(k)=\log\frac{\text{E}(W_{k}^{R})}{\text{E}(W_{1}^{R})}-\log\frac{W_{k}}{W_{1}},$$

where E($W_{k}^{R}$) is the expectation of the WSS for the reference dataset, which has no clusters. Tibshirani *et al*. [11] suggested using a uniformly distributed reference dataset. E($\sum_{k}\delta_{k}=1$) is estimated by randomly generating *B *uniformly distributed datasets.

$$\hat{\text{E}}(W_{k}^{R})=\frac{1}{B}\sum_{b=1}^{B}W_{k}^{R(b)}$$

We then estimate the number of clusters by finding the smallest *K *such that

(6) $$\text{Gap}(K)\geq \text{Gap}(K+1)-{s^{\prime }}_{K+1},$$

where ${s^{\prime }}_{K+1}=s_{K+1}\sqrt{1+\frac{1}{B}}$ and *s*_*K*+1 _is the standard error of $W_{k+1}^{R}$. The gap statistic can be used with any clustering algorithm. In this study, we use it along with K-means to predict the number of clusters. It is generally the case that we can better fit a dataset to the model with more parameters, resulting in higher likelihood or lower sum of squared error. Therefore, the BIC score addresses this issue by penalizing the number of parameters. It is defined as

BIC = 2L(θ*) - log(*m*)|θ*|,

where L is the log likelihood function, θ* is the parameter set maximizing the likelihood and *m *is the number of observations or samples. The BIC score is used in MCLUST Version 3 [8] as the model selection criterion.

### Evaluation metric

In population structure inference, given the number of clusters, each individual in the dataset is assigned an estimated membership coefficient for each cluster. The coefficient indicates the likelihood that an individual descends from a specific population origin. By assigning each individual to the most likely cluster, we have obtained a partition of the individuals in a dataset. A partition is a set of mutually exclusive and collectively exhaustive clusters. Given two partitions, we use the algorithm proposed by Konovalov *et al*. [13] to measure the distance between them. The distance between two partitions is defined as the minimum number of individuals that need to be removed from each partition in order to make the two partitions identical. For clarity, we scale the distance measure to [0, 1].

For the simulated datasets, we calculate the distance between the gold-standard partition and the partition generated by each clustering algorithm. The smaller the distance between the two partitions, the better the performance. For the real datasets, we compare the partition produced by STRUCTURE to the partitions produced by all other clustering algorithms investigated in this study. This is because STRUCTURE is a widely used algorithm in inferring population structure.

## Results and discussion

Table 2 shows the number of significant PCs selected for each dataset using the TW statisitc at *p*-value = 0.05. We can see that PCA reduces the number of variables from around 5,000 to at most 70. However, we suspect that there are still noisy and non-informative PCs hidden in those selected significant ones. Therefore, we are also interested in using only the top-3 PCs with the largest eigenvalues. We then perform cluster analyses on the reduced datasets using those generic algorithms described in Sectoin **Methods**. The results are shown in the following subsections.

**Table 2:** Number of principal components selected using TW statistic at *p*-value = 0.05. The simulated datasets are denoted as s1 through s4.

Set	close	dist	s1	s2	s3	s4
#PCs	15	70	2	4	18	3

### Simulated Data

**Figure 3:**
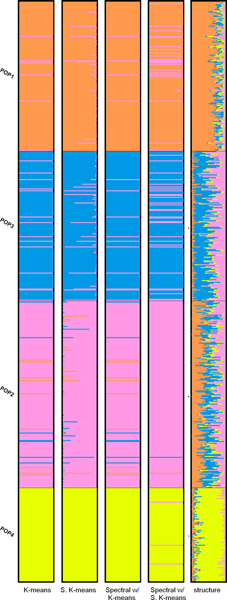
Bar plots of results of the fourth simulated dataset (*K *= 4).

**Figure 4:**
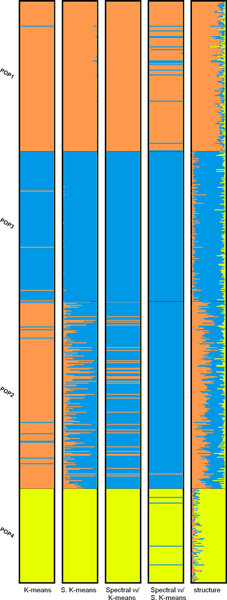
Bar plots of results of the fourth simulated dataset (*K *= 3).

Evaluating the performance of the clustering algorithms on simulated datasets is straightforward since the gold standard partition for each dataset is available. The performance, in terms of distance between the gold standard partition and the predicted one, is summarized in Table 4. The measure of distance is described in Section **Methods**. The parameter *γ *in Equation 4 is not tuned for all the simulated datasets. It is set to either 1 or $\frac{1}{2}$, except for the third dataset. The reason for setting *γ *= 2^-4 ^is because when the algorithm tries to obtain the eigenvalues and eigenvectors of $D^{-\frac{1}{2}}(D-S)D^{-\frac{1}{2}}$ (as described in Figure 2) the R function eigen seems to be caught in an infinite loop for *γ *= 2^-*g*^, *g *∈ {0, 1, 2, 3}. For the first two datasets, all the clustering algorithms show perfect results. This is probably because these two datasets contain independent and equal-sized subpopulations. For the third dataset, apart from the two variants of spectral clustering algorithm, soft K-means and STRUCTURE perform equally well while K-means produces comparable results. Moreover, soft K-means performs the best on the fourth dataset while STRUCTURE gives the worst performance. To better analyze the results, we visually compare the clustering algorithms using bar plots shown in Figure 3. The bar plots are generated using software DISTRUCT [14]. According to the demography in Figure 1, population 3 does not contain admixed individuals but STRUCTURE fails to assign the individuals in population 3 to only one cluster as the other algorithms do. However, when setting *K *= 3, STRUCTURE performs very well and reflects the demography used to simulate the data. The bar plots are shown in Figure 4. We can see that individuals in population 1, 3 and 4 are clustered into distinct groups, while individuals in population 2 equally likely belong to the two clusters occupied by population 1 and 3. Soft K-means produces similar results, while the other algorithms group individuals in population 2 with individuals in either population 1 or population 3. Table 3 shows the number of clusters inferred by the gap statistic, the BIC score and STRUCTURE. We can see that the BIC score with PCs suggested by the TW distribution and STRUCTURE make identical predictions on the simulated datasets. When the BIC score is used with the top-3 PCs, it makes the correct prediction on the second simulated dataset but fails on the third one. Therefore, these two approaches perform comparably on the simulated datasets. The gap statistic fails to make the correct prediction on all but the first simulated dataset unless only 2 or 3 PCs are used.

**Table 3:** Predicted number of clusters for each dataset

Set	close	dist	s1	s2	s3	s4
True *K*	NA	NA	3	4	4	4
Gap	1	7	**3**	1	1	1
	1^1^	1^1^	--	**4**^1^	**4**^3^	**4**^2^
BIC	3	3	**3**	5	**4**	**4**
	3^1^	6^1^	--	**4**^1^	6^1^	**4**^1^
STRU^4^	6	6	**3**	5	**4**	**4**

^1 ^3 PCs. ^2 ^2^nd ^*K*. ^3 ^3PCS, 2^nd ^*K*. ^4 ^STRUCTURE.

**Table 4:** Results on the simulated datasets in terms of distance

Set	K^1^	SK^2^	SpK^3^	SpSK^4^	STRU^5^
1	**0**	**0**	**0**^6^	**0**^6^	**0**
2	**0**	**0**	**0**^7^	**0**^7^	**0**
3	0.01	**0**	0.598^8^	0.596^8^	**0**
4	0.058	**0.034**	0.048^7^	0.089^7^	0.342

^1^K-means. ^2^Soft K-means. ^3^Spectral + K-means. ^4^Spectral + Soft K-means. ^5^STRUCTURE. ^6 ^*γ *= $\frac{1}{2}$. ^7 ^*γ *= 1. ^8 ^*γ *= 2^-4^.

### Real Data

In this section, we compare the results generated by the generic clutering algorithms to those produced by STRUCTURE since no gold standard partitions are available for the real datasets. The results for the distant and close dataset are shown in Table 5 and Table 6, respectively. For the distant dataset, using all the 70 significant PCs, the partition given by soft K-means at *K *= 2 is identical to that produced by STRUCTURE. When only the top-3 PCs are used, all the clustering algorithms produce partitions similar to that predicted by STRUCTURE. This implies that all the distance-based generic algorithms investigated in this study are sensitive to noisy and non-informative variables, which are used in the calculation of distance or similarity.

**Table 5:** Comparison of the results on the distant dataset with STRUCTURE

K	#PCs	K^1^	SK^2^	SpK^3^	SpSK^4^
2	70	0.252	**0**	0.137^5^	0.103^6^
2	3	0.03	**0.003**	0.004^7^	0.019^7^
3	70	0.3	0.101	0.422^8^	0.349^9^
3	3	**0.042**	**0.045**	**0.041**^7^	0.123^7^
4	70	0.401	0.617	0.414^8^	0.433^10^
4	3	0.304	0.277	0.311^7^	0.337^7^

^1^K-means. ^2^Soft K-means. ^3^Spectral + K-means. ^4^Spectral + Soft K-means. ^5^*γ *= 2^-5^. ^6^*γ *= 2^-1.5^. ^7^*γ *= 1. ^8^*γ *= 2^-6.5^. ^9^*γ *= 2^-6^. ^10^*γ *= 2^6^

**Table 6:** Comparison of the results on the close dataset with STRUCTURE

K	#PCs	K^1^	SK^2^	SpK^3^	SpSK^4^
2	15	0.415	0.372	0.337^5^	0.31^6^
2	3	**0.109**	0.194	0.252^5^	**0.101**^5^
3	15	0.512	0.271	0.403^7^	0.353^8^
3	3	0.36	0.252	0.298^5^	0.384^5^
4	15	0.554	0.558	0.473^9^	0.376^10^
4	3	0.426	0.419	0.481^5^	0.523^5^

^1^K-means. ^2^Soft K-means. ^3^Spectral + K-means. ^4^Spectral + Soft K-means. ^5^*γ *= 1. ^6^*γ *= 2^-2.5^. ^7^*γ *= 2^-2^. ^8^*γ *= 2^-6.5^. ^9^*γ *= 2^-4.5^. ^10^*γ *= 2^-4^

**Figure 5:**
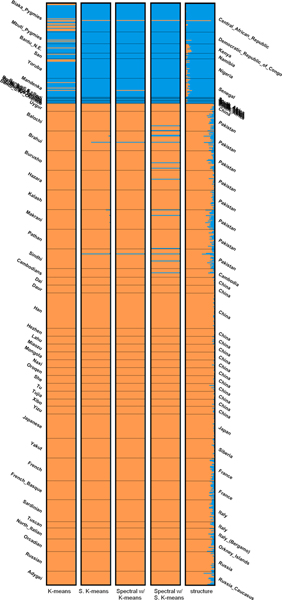
Bar plots of results of the distant dataset (*K *= 2).

**Figure 6:**
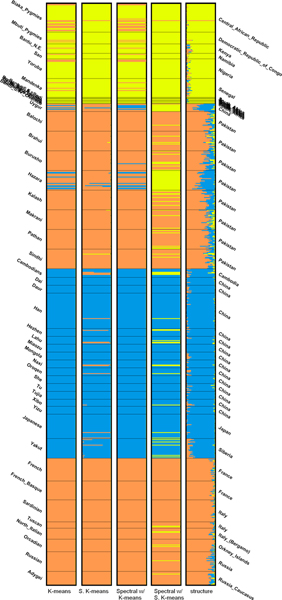
Bar plots of results of the distant dataset (*K *= 3).

**Figure 7:**
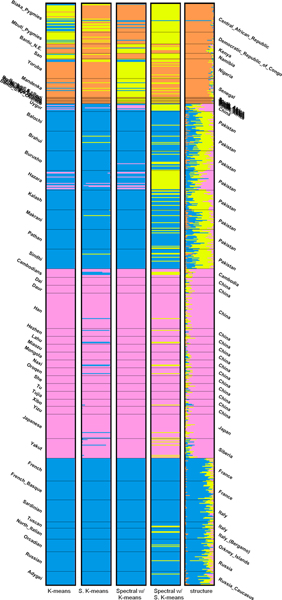
Bar plots of results of the distant dataset (*K *= 4).

The bar plots of the partitions produced using the top-3 PCs are shown and compared to the one by STRUCTURE in Figure 5. We can see that the populations in Africa are grouped into one cluster and all the other populations are grouped into the other one. This phenomenon is more evident when *K *= 3. As seen in Table 5, the partitions produced by the generic algorithms using 3 PCs are more similar to the one produced by STRUCTURE than those produced using 70 PCs. The bar plots are shown in Figure 6. For *K *= 4, however, the partitions generated by the generic clustering algorithms are very different from that by STRUCTURE. Using the top-3 PCs hardly makes the distance smaller. From the plots in Figure 7, we can see that STRUCTURE infers that the genome of individuals in Pakistan is the mixture of the blue, yellow and pink clusters and the yellow one makes the most contribution. The other algorithms group the individuals in Pakistan and Europe into the same cluster.

**Figure 8:**
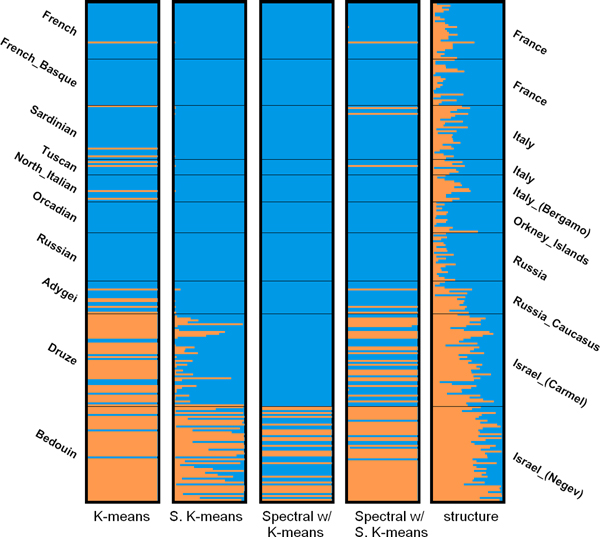
Bar plots of results of the close dataset (*K *= 2).

**Figure 9:**
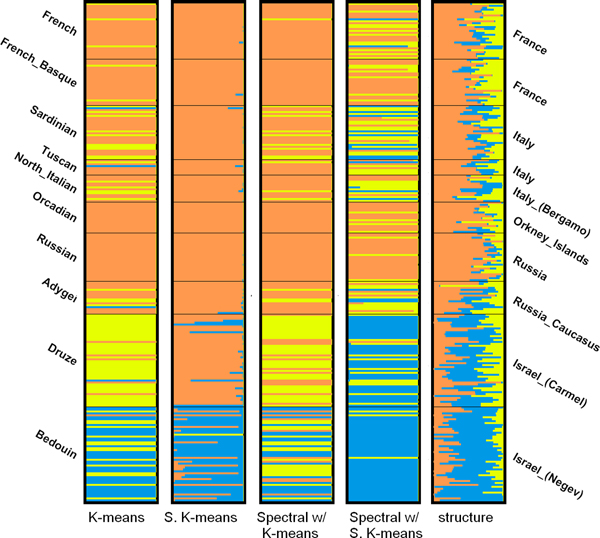
Bar plots of results of the close dataset (*K *= 3).

As for the close dataset, it can be seen in Table 6 that K-means and spectral clustering with soft K-means produce the most similar partitions to the one generated by STRUCTURE at *K *= 2 using the top-3 PCs. The bar plots for *K *= 2 and *K *= 3 using 3PCs are shown in Figure 8 and 9, respectively. When *K *= 2, K-means groups almost all the individuals in Israel into one cluster and groups the rest into the other cluster, which is very similar to the results given by STRUCTURE. At *K *= 3, although K-means does not produce the most similar partition, it subdivides the individuals in Israel into two clusters, which correspond to the Druze and Bedouin populations. We can also observe a similar pattern in the bar plot produced by STRUCTURE. The individuals in the Bedouin population generally have a higher proportion of genome from the blue cluster than the individuals in the Druze population, enabling us to distinguish between the two populations.

It is difficult if not impossible to assess the correctness of the predicted number of clusters for the real datasets. We can see in Table 3 that, the three methods give completely different predictions on the two real datasets. STRUCTURE suggests that there are 6 clusters in the close dataset. However, the bar plot (not shown) at *K *= 6 is very noisy and does not reveal 6 clusters in the population. The BIC score predicts 3 clusters in the close dataset. The bar plot generated by soft K-means at *K *= 3 in Figure 9, however, is not convincing, since only one individual is assigned to the yellow cluster. STRUCTURE and the BIC score (with 70 PCs) suggest 6 and 3 clusters, repectively. Three clusters seem reasonable according to the bar plots in Figure 6. However, we can not observe 6 clusters in the bar plots generated by STRUCTURE at *K *= 6 (not shown). For both real datasets, the likelihood given by STRUCTURE increases as *K *increases, which is a sign of over-fitting. The gap statistic seems to suffer from the presence of noisy and non-informative PCs and either predicts no structure (*K *= 1) or a large *K *of 7, which is not supported by the bar plot (not shown).

## Conclusion

In this study, we investigated three generic clustering algorithms on genotype data. We applied PCA to genotype data in order to reduce the number of variables. Based on the TW-statistic, the significant PCs were kept for subsequent cluster analyses. A *p*-value of 0.05 was used in selecting significant PCs. We showed that all the generic clustering algorithms perform as well as STRUCTURE on the first three simulated datasets. Moreover, for the fourth dataset, all these algorithms produce better partitions than the one predicted by STRUCTURE. We showed that soft K-means and K-means perform comparably well to STRUCTURE on the distant and close datasets, respectively. However, all the three generic clustering algorithms show different degrees of susceptibility to noisy and non-informative PCs. Therefore, the choice of *p*-value remains an important issue.

We also showed that STRUCTURE and the BIC score produce identical predictions on the simulated datasets. When it comes to real datasets, STRUCTURE predicts the number of clusters to be the largest *K *investigated, showing a sign of over-fitting. The BIC score is, therefore, a better index in predicting the number of clusters for real datasets, which reinforces the finding by Zhu *et al*. [15]. The gap statistic performs poorly due to the presence of non-informative PCs.

While STRUCTURE is a sophisticated clustering algorithm designed for genotype data, it is very time-consuming because of the nature of MCMC. We believe that the choice of clustering algorithms depends on the purpose of population structure inference. If we want to infer recent demographic events, STRUCTURE would be a good choice since it even considers the origin of an alelle copy in the model. However, if population structure inference is used as a preprocessing step in association studies, PCA with soft K-means would be very handy. In stratified association study, we need sufficient individuals in each cluster to make significant and meaningful associations. Hence, splitting two slightly different populations and thus making each cluster smaller may not be helpful to association studies.

Based on the results of this study, we recommend choosing suitable clustering algorithms according to the nature of applications of population structure inference. In addition to the proper choice of *p*-value in selecting PCs, we recommend applying unsupervised feature selection algorithms, such as the one proposed by Paschou *et al*. [16], to genotype data to improve the stability and robustness of the combination of PCA and a generic clustering algorithm.

## Competing interests

The authors declare that they have no competing interests.

## Authors' contributions

CL conceived the study, collected the real data, carried out the implementation, conducted cluster analyses with the generic clustering algorithms and drafted the manuscript. AA conducted the STRUCTURE experiments. CH guided the study and revised the manuscript. All authors read and approved the final manuscript.
